# Unveiling the Hidden Drivers: How Vegetation Cover, Season and Forest Management Shape the Soil Microbial Community in Two Mediterranean Forest Ecosystems

**DOI:** 10.1111/1758-2229.70255

**Published:** 2026-03-19

**Authors:** Enrica Picariello, Flavia De Nicola

**Affiliations:** ^1^ Department of Sciences and Technologies University of Sannio Benevento Italy

**Keywords:** CLPP, DNA analyses, enzyme activity, forest soil microbiome, interactive effect, microbial community function, microbial community structure

## Abstract

Soil provides essential ecosystem services and serves as a habitat for biodiversity, but it is often affected by disturbances from management practices and seasonal changes, which can alter its microbial communities. This study investigated the combined effects of dominant vegetation, forest management, and seasonal variation on soil microbial communities and enzyme activity over one year in turkey oak and beech forests managed as high forest or coppice. Results showed that the dominant vegetation type had a greater influence on microbial communities than seasonal changes. While forest management did not significantly affect microbial activity, it altered microbial community composition. In beech forests, bacterial communities (at the order level) showed relative abundances higher in soil under high forest with respect to coppice, whereas the fungal community showed orders most abundant under coppice management with respect to the high forest. Forest management changed the relative abundances of microbial communities, but it did not remarkably affect microbial community functions and, thus, the associated ecosystem services. Our results highlight that the forest type should be considered when evaluating forest management. This study offers new insights into the factors influencing the composition of soil microbial communities and their associated ecosystem functions.

## Graphical abstract

Forest management affects microbial community composition depending on forest type. Vegetation type has the strongest effect on microbial composition, while management alters community structure without affecting function, highlighting the importance of vegetation in forest soil ecosystem dynamics.

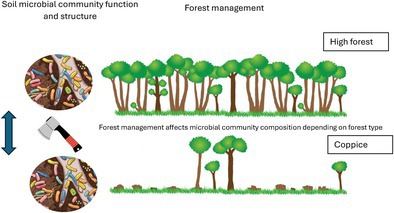

## 1 Introduction

Soil provides essential ecosystem services, supports biodiversity, aids carbon sequestration, and regulates water and biochemical element cycles. However, growing human activity is threatening soil health (Lawler et al. 2014; Rillig et al. 2023), resulting in a sharp decline in these services (Pereira et al. 2018).

Soil plays a crucial role in the functioning of terrestrial ecosystems; however, human activities cause disturbances that can drive changes in soil microbial community structure and function (Lawler et al. 2014; Baiano et al. 2024; Picariello et al. 2024). Also, forest management practices, that is any human action related to timber production, such as coppicing, can be considered disturbances to ecosystems (Picariello and De Nicola 2024). While high‐intensity management results in a significant supply of timber biomass, it has detrimental effects on soil biodiversity and health (Sing et al. 2017). These practices can alter the natural forest composition by removing tree species, changing the age‐class structure, exporting biomass, and reducing the amount of dead wood (Paillet et al. 2010). Additionally, cutting alters light and microclimate conditions, which, in turn, affect the undergrowth and the soil properties. In response to management practices, new microhabitats form, often leading to shifts in microbial communities (Drenovsky et al. 2010; Ananbeh et al. 2019; Venanzi et al. 2020).

In this context, the European Union has defined mitigation measures to reduce the effects of forest management practices and address many challenges, such as increasing carbon storage through sustainable management practices (Regulation (EU) 2018). Among the main sustainable forest management practices employed in Europe are selective cutting, which involves the removal of specific trees, typically mature or over‐mature, while preserving most of the forest. This approach maintains structural diversity, minimizes ecological disturbance, and supports natural regeneration (Putz et al. 2022). Another key practice is Continuous Cover Forestry, which avoids traditional clear‐cutting, maintains continuous forest cover, and promotes diverse age structures, enhanced biodiversity, and improved microclimatic conditions (Ersson et al. 2023).

Vegetation cover, soil properties, and microbial communities are closely interconnected. The vegetation cover affects the quality of the litter, which, in turn, shapes microbial community composition and function (Picariello et al. 2021). Additionally, tree roots play a crucial role in supplying soil microorganisms with easily accessible carbon through exudates, influencing the structure and function of the microbial community (Liu et al. 2018; Tian et al. 2018). Alterations in the structure or function of microbial communities can affect the turnover and stabilisation of soil organic matter (SOM) as well as ecosystem processes (Prommer et al. 2019).

Seasonality greatly affects the composition of the soil microbial community by influencing both biotic and abiotic factors. In temperate ecosystems, ecological factors such as temperature, moisture, and nutrient availability vary throughout the year. Seasonal changes in plant activity and carbon inputs also can impact soil organic carbon and microbial biomass carbon (Waldrop and Firestone 2006; Babur and Dindaroglu 2020; Ramírez et al. 2020).

Forest management practices, climatic conditions, and vegetation cover interact to shape the ecological dynamics of the soil microbial communities (Felsmann et al. 2015; Bastida et al. 2017, 2019; Luo et al. 2020). Despite growing recognition of these interactions, few studies have clearly distinguished which of these factors exerts the predominant influence on soil microbial community structure and function (Han et al. 2021).

To address these knowledge gaps, this study aims to:

(1) Evaluate the combined effect of forest system (turkey vs. beech), and thus of different vegetation cover, forest management practices (coppice vs. high forest) and seasonality on the functional and structural diversity of the soil microbial community of the most representative forests of the Mediterranean area;

(2) determine the relative contributions of these factors to changes in microbial community structure and function.

We hypothesized that: (i) the dominant tree cover would exert a stronger influence on soil microbial community structure and functions than forest management (due to the long interval elapsed since cutting) or seasonal variability, due to differences in litter quality, root exudation, and organic matter input; (ii) forest management would alter the relative abundance of microbial taxa and microbial functions.

The innovative aspect of this work lies in its integrative approach: by simultaneously analysing vegetation cover, forest management, and seasonal variability, we try to clarify their relative contributions to both microbial community structure and functions. While previous studies have generally investigated these drivers separately (Bastida et al. 2019; Nocentini et al. 2022), few have compared their combined and interactive effects in Mediterranean forests, despite their ecological importance.

## 2 Materials and Methods

### 2.1 Study Area and Sampling

This study focuses on widespread forest types in Europe: European beech ( *Fagus sylvatica* ) and Turkey oak ( *Quercus cerris* ) forests, covering about 14 and 3 million hectares, respectively, according to a publication by EUFORGEN (European Forest Genetic Resources Programme 2011) and European Commission (2006). These forest types represent 0.24% (beech) and 1.18% (turkey oak) of the total area of the Italian forests (Gasparini et al. 2022).

Beech and turkey oak woodlands were chosen in the Matese area on lithologically similar sites, with the substrate classified as Pachi‐Eutrisilic Andosol (FAO 1976). According to Shepard's classification, the soil texture was silty sand beneath beech trees and sandy silt beneath turkey oak trees (Shepard 1954).

**FIGURE 1:**
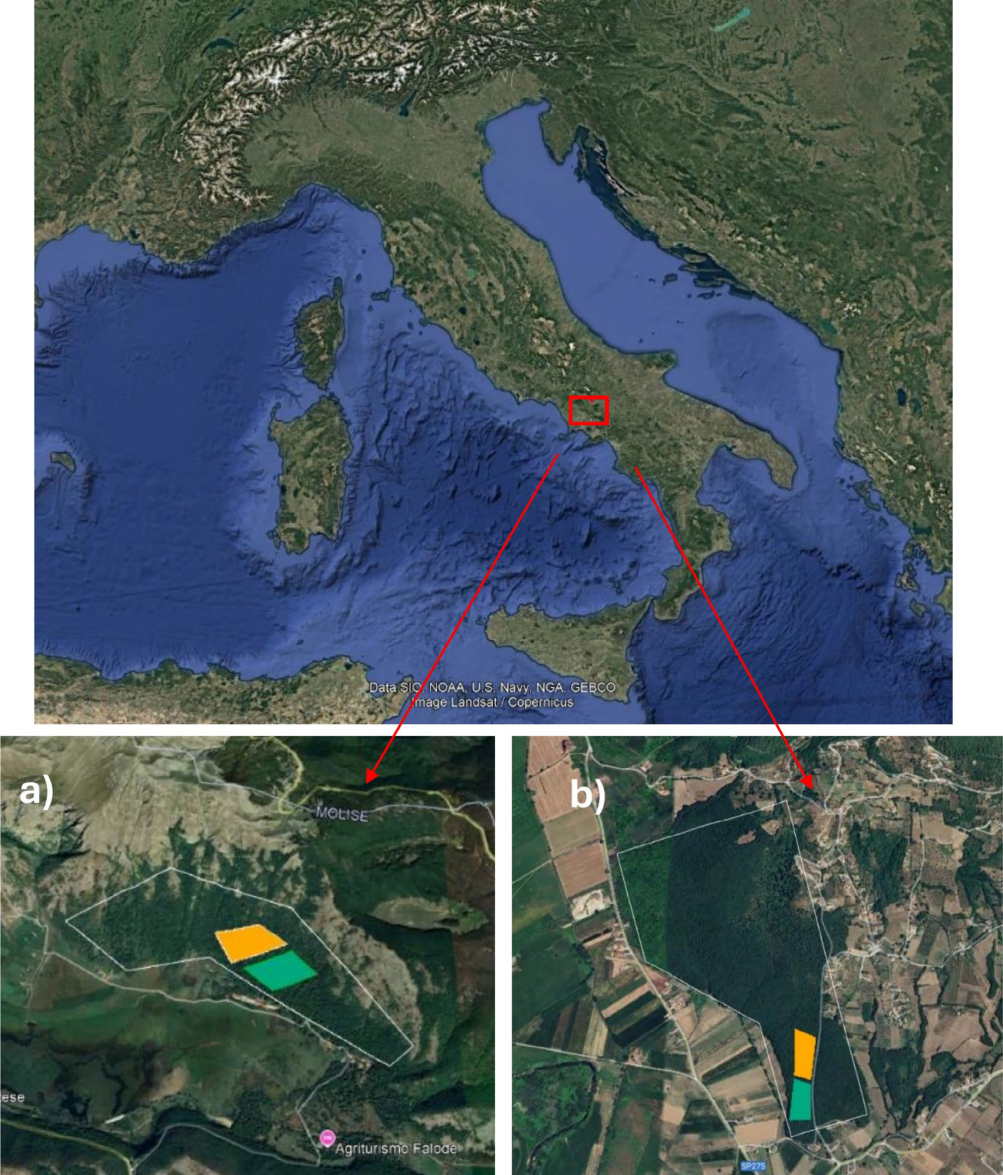
Map of the study area (Matese mountains, Southern Italy) with the indication of the polygons in (a) beech (geographical coordinates: 41°25′00.5”N; 14°25′53.2″ E) and (b) turkey oak (geographical coordinates: 41°23′06.2”N; 14°08′24.6″ E). Green = high forest, orange = coppice.

For each forest type, we selected two adjacent areas with different management practices (Figure 1): one forest was managed through coppicing, with the last cut occurring at the beginning of the 21st century, while the other has remained unmanaged since the early 20th century and has since developed into high forest. In each forest, a completely randomised design was applied, comprising six plots per management type, covering a total area of 150 m^2^. In each plot, ten soil cores were randomly collected, according to a zigzag pattern, from a depth of 0 to 10 cm and subsequently pooled to create a uniform and representative sample. The samplings were carried out over one year in summer, autumn, winter and spring. This resulted in a total of 48 soil samples collected. The planned experimental design and sampling strategy were adequate for capturing coppicing, seasonality and cover effects (as tested by ω^2^ explained below in the data analysis).

### 2.2 Laboratory Analyses

#### 2.2.1 Soil Physico‐Chemical Analysis

Soil chemical analyses were conducted following the methods outlined by Colombo and Teodoro (2015). Water content (WC) was determined by the drying method (105°C until constant weight) on sieved (< 2 mm) soil samples. Soil pH was measured using the potentiometric method (SenseION + PH3, Hach) in a water suspension (1:5, w/v soil: water). Soil organic matter (SOM) was assessed through calcination in a muffle furnace (550°C for 4 h; Nabertherm GmbH, Controller B 170) and converted to soil organic carbon (SOC) using a conversion factor (Pribyl 2010). Labile (Cl) and recalcitrant (Cr) soil organic carbon were extracted using an acid solution (H_2_SO_4_) (Ahmed et al. 2016). Labile and recalcitrant soil carbon were quantified using spectrophotometric analysis at 254 nm (UV–VIS Jenway 6715 Spectrophotometer), following a modified version of Standard Methods 5910 B (SM 2000). Each sample was analyzed in triplicate.

#### 2.2.2 Enzyme Activities

Six enzymatic activities were analyzed by spectrophotometric method: hydrolase activity (Schnurer and Rosswall 1982), arylsulfatase activity (Dick et al. 1996), β‐glucosidase activity (Rodríguez‐Loinaz et al. 2008), laccase activity (Floch et al. 2007), phosphatase activity (Dick et al. 1996) and β‐glucosaminidase activity (Parham and Deng 2000). In brief, 0.1 g of sieved (< 2 mm) soil was incubated for 30 min with the respective substrates, under shaking, at the optimal pH and temperature for the specific enzymatic reaction. Hydrolase activity was assessed using a fluorescein calibration curve at 490 nm. The activities of arylsulfatase, β‐glucosidase, phosphatase, and β‐glucosaminidase were quantified based on a p‐nitrophenol calibration curve at 410 nm. Laccase activity was determined by measuring the absorbance of the reaction product (ABTS^+^) at 420 nm. All analyses were performed in triplicate for each sample.

#### 2.2.3 Functional Diversity

Microbial functional diversity, based on carbon utilization patterns (community‐level physiological profiling, CLPP), was evaluated using the Biolog EcoPlate method. The procedure was conducted as outlined by Feigl et al. (2017), with some modifications (Xu et al. 2015). Four grams of soil were suspended in 36 mL of a 0.85% sterile NaCl solution and shaken for 30 min. A serial dilution was prepared using the same NaCl solution, and 150 μL of the 10^−3^ dilution was dispensed into each well of the EcoPlate, which was subsequently incubated at 25°C. Absorbance was recorded every 24 h over a 192‐h period at 600 nm using a Bio‐Rad Microplate Reader (Model 680). Each sample was analysed in triplicate.

To address the limitation that fast‐growing microbes can disproportionately influence CLPP results, we normalised the absorbance data by the Average Well Colour Development (AWCD), ensuring that comparisons among samples account for differences in overall metabolic activity.

#### 2.2.4 DNA Analyses

Total soil DNA was extracted using a GENELUTE (TM) SOIL DNA ISOLATION KIT (Sigma Aldrich), following the manufacturer's instructions. DNA yield (ng DNA g^−1^ soil d.w.) and purity were evaluated using Nanodrop One (Thermo Scientific), and DNA quality was assessed by agarose gel electrophoresis.

PCR amplification of the target regions was carried out using specific primers linked to barcodes. The V3–V4 regions of the bacterial 16S rRNA gene were amplified using primer sets 341F (CCTAYGGGRBGCASCAG) and 806R (GGACTACNNGGGTATCTAAT) and the ITS region was amplified using the fungal primer sets of ITS1‐1F‐F (CTTGGTCATTTAGAGGAAGTAA) and ITS1‐1F‐R (GCTGCGTTCTTCATCGATGC).

Each sample was assigned a unique eight‐base barcode sequence. PCR reactions were performed in a total volume of 15 μL, containing Phusion High‐Fidelity PCR Master Mix (New England Biolabs), 0.2 μM of each forward and reverse primer, and approximately 10 ng of template DNA. The thermal cycling protocol included an initial denaturation at 98°C for 1 min, followed by 30 cycles of denaturation at 98°C for 10 s, annealing at 50°C for 30 s, and extension at 72°C for 30 s, with a final elongation step at 72°C for 5 min. PCR products of the correct size were selected using 2% agarose gel electrophoresis and purified through magnetic bead purification. The samples were mixed in equal density ratios according to the concentration of PCR products. After thorough mixing, the PCR products were analyzed, and the target bands were recovered.

Equal amounts of PCR products from each sample were pooled, end‐repaired, A‐tailed, and then ligated with Illumina adapters. The libraries were sequenced using a paired‐end Illumina platform, producing 250 bp paired‐end raw reads. Quantification was performed with Qubit, real‐time PCR was used to assess library concentration, and a bioanalyzer was employed to determine size distribution. The quantified libraries were then pooled and sequenced on the Illumina NovaSeq 6000 platform (PE250), according to the required effective concentration and data output. Amplicon library preparation, sequencing, and bioinformatics analysis services were provided by Novogene Co. Ltd. (Beijing, China).

### 2.3 Data Analysis

The Biolog eco‐plates data were used to calculate the average well colour development (AWCD) with the formula proposed by Garland and Mills (1991).

To evaluate the combined effects of forest type, management, and season (treated as fixed factors) on soil characteristics, we performed a permutational multivariate analysis of variance (PERMANOVA). The analysis included both chemical parameters (pH, water content, soil organic matter, total organic carbon, labile and recalcitrant carbon fractions) and biological parameters (activities of six enzymes: hydrolase, arylsulfatase, β‐glucosidase, laccase, phosphatase, β‐glucosaminidase, together with community‐level physiological profiling indices such as AWCD). The analysis is specifically suited for multivariate ecological datasets, allowing the analysis of community‐level responses through dissimilarity matrices and permutation‐based testing, without assuming multivariate normality. To address multicollinearity, highly correlated, non‐numeric, missing, or constant variables were removed, and the remaining variables were standardized before PERMANOVA. PERMANOVA *p*‐values were obtained using a Monte Carlo permutation test with 999 permutations. Pairwise comparisons between factor levels were performed using the pairwise Adonis R package, with *p*‐value adjustments to account for multiple testing. Additionally, omega‐squared (ω^2^) was calculated to ensure that the sampling strategy was able to capture both the coppicing effect and the season effect.

Non‐metric multidimensional scaling (NMDS) was employed to represent the multivariate separation among groups defined by forest type, management, and season, with superimposed 95% confidence ellipses.

Variation partitioning procedure (Borcard et al. 1992) was used to establish relationships between forest type/season and forest management/season.

The total variance in enzyme activities and relative abundance at the order level was explained by conducting a full LDA, using the two groups of environmental variables together as constraining explanatory factors. A Venn diagram was created to visualize the different portions of the total variance.

Differences in bacterial and fungal community structure between forest type and forest management were processed on standardised data using two separate Q‐type principal component analyses (PCA), analyzing soil physico‐chemical characteristics and the relative abundance of bacterial and fungal orders in the two forest systems.

The “vegan,” “tidyverse,” “nlme,” “ggplot2,” “plotrix,” “nlme,” “vegan,” “pairwiseAdonis,” “candisc,” “ggsci,” “ellipse,” “factoextra,” “GGally” and “car” packages in the R 4.1.2 programming environment (R Core Team 2021) were used.

The barplots, relative to molecular analysis data, showing the relative abundances of bacterial and fungal orders for each sample, were created using Perl software 5.26.2 with the SVG function.

## 3 Results

The PERMANOVA analysis (ω^2^ = 0.94) on chemical parameters, enzymatic activities, and carbon consumption patterns revealed significant differences between forest systems (F = 14, *p* < 0.001) as well as across seasons (F = 48, *p* < 0.001) but none between forest management (Table S1).

**FIGURE 2:**
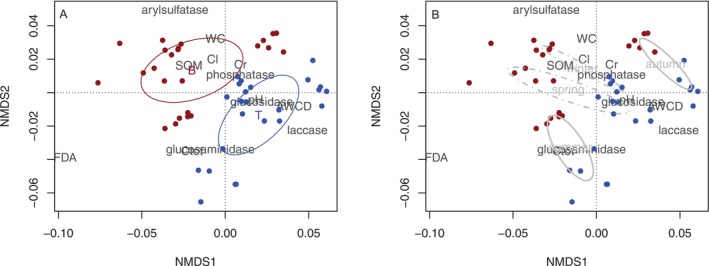
Non‐metric multidimensional scaling (NMDS) biplot, with the superimposition of the confidence ellipses (α = 0.05), showing the differentiation (a) between forest systems (beech = B and turkey oak = T) and (b) among seasons in relation to physico‐chemical properties and enzymatic activities measured.

The separation between forest systems was mainly explained by differences in soil chemical–physical properties (Figure 2a). Beech soils had higher water content (45% vs. 25%), soil organic matter (26% vs. 13%), total organic carbon (190 vs. 160 mg C g^−1^ d.w.), and labile carbon (30 vs. 17 mg C g^−1^ d.w.) compared to turkey oak soils. Soil pH was neutral under turkey oak (pH 7) and slightly acidic under beech (pH 6) (Tables S2 and S3). In contrast, seasonal separation (Figure 2b) was mainly associated with enzymatic activities and CLPP. Arylsulfatase activity was higher in spring and winter than in summer and autumn, whereas laccase, glucosidase, phosphatase and AWCD reached their maximum values in autumn.

**FIGURE 3:**
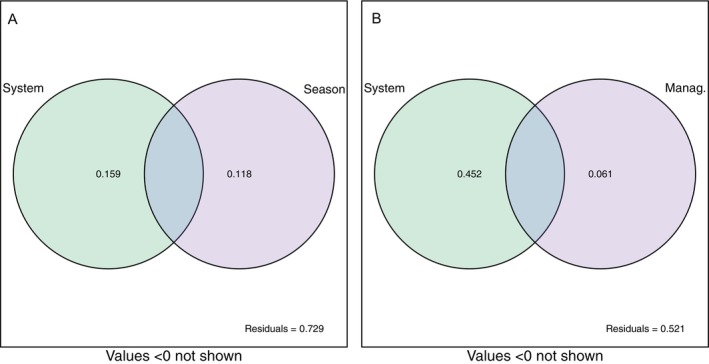
Venn diagram showing the variance partitioning by (a) forest system and season on enzymatic activities and (b) forest system and management on microbial community structure at order level. Percentages of explained variances are indicated.

The variation partitioning (Figure 3a) showed that forest type and season explained 16% and 12% of the variance in microbial community function, respectively. In terms of community structure, forest type accounted for 45% of the variance, whereas management explained 6% (Figure 3b).

**FIGURE 4:**
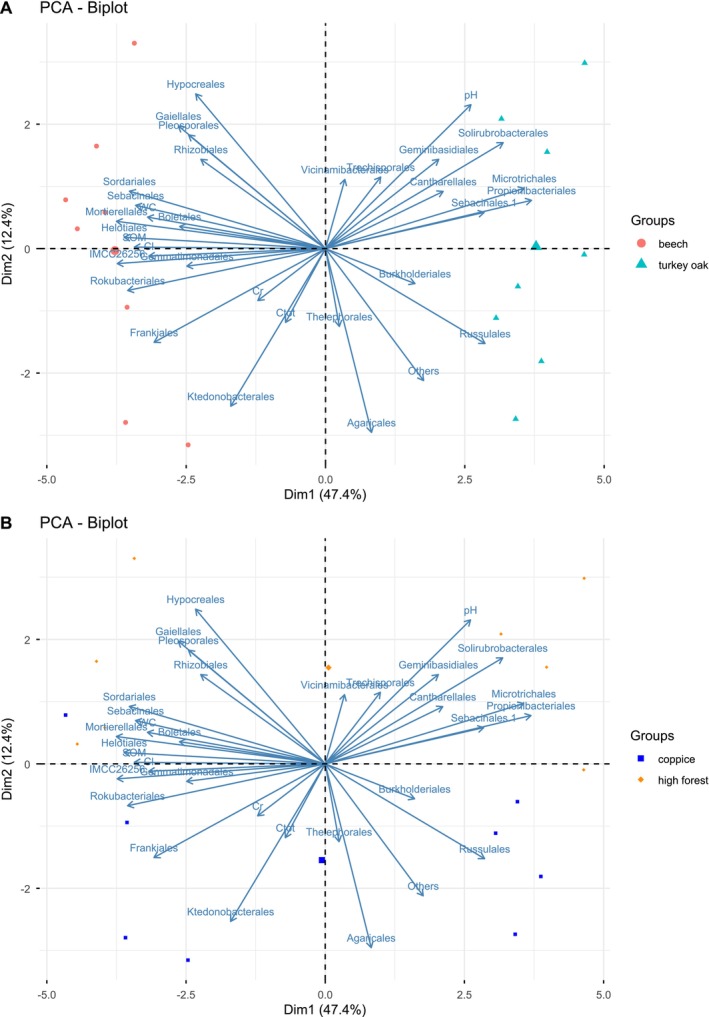
Biplots of principal component analyses (PCA) showing the differentiation between (a) forest systems and (b) forest management, based on soil physico‐chemical characteristics and the microbial community structure at order level. The plots display the first (Dim1) and second (Dim2) principal components and the variance explained by Dim1 and Dim2.

PERMANOVA performed on chemical parameters and the relative abundance of bacterial and fungal communities at the order level revealed significant differences between forest systems (F = 26, *p* < 0.001) and forest management (F = 2, *p* < 0.014), but not among seasons. The PCA of bacterial and fungal community structure showed that Dim1 (47.4% of variance) separated the two forest systems (Figure 4a), while Dim2 (12.4% of variance) separated management types (Figure 4b). The microbial community showed a different structure in the two forest soils. The separation between forest systems was driven by the higher SOM, Cl, WC content (Tables S2 and S3) and the higher relative abundance of the bacterial order *Frankiales (*0.04 vs 0.02%*)* found in soil under beech with respect to turkey oak (Figure S1a). Conversely, turkey oak soils were characterised by the higher relative abundances of the bacterial order *Solirubrobacterales* (0.1% vs 0.04%) and the fungal order *Russulales* (0.3% vs 0.1%) with respect to beech.

Although overall community structure did not differ between management types, several bacterial and fungal orders showed distinct relative abundances. The separation between the two forest managements was driven by the higher soil pH and higher relative abundance of *Agaricales* (0.3% vs. 0.1%) in areas managed with coppicing (Figure S1b), and the presence of a higher relative abundance of fungal orders, such as *Hypocreales* (0.08% vs. 0.05%), *Geminibasidiales* (0.1% vs. 0.05%) and *Pleosporales* (0.1% vs. 0.03%) in soil under high forest with respect to coppice.

**FIGURE 5:**
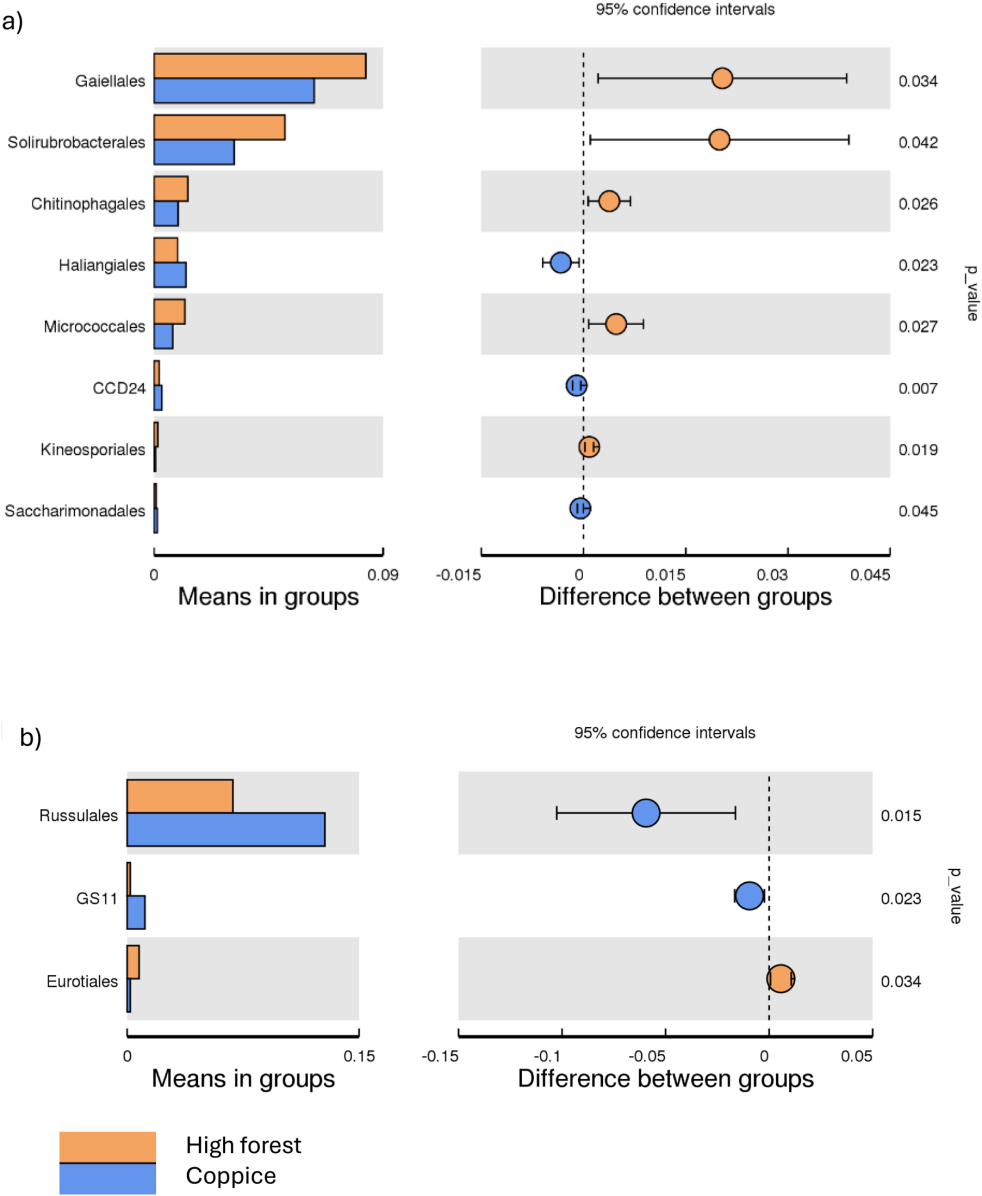
Barplots (left panel) showing the relative abundances (%) of soil (a) bacterial and (b) fungal orders in beech forests managed by coppice and high forest. The right panel displays the difference in relative abundances of each order between the two management types (± lower and upper limits of 95% confidence interval).

In beech forests, soil bacterial and fungal communities respond differently to management practices. The relative abundance of bacterial orders such as *Gaiellales* (0.08% vs. 0.06%), *Solirubrobacterales* (0.05% vs. 0.03%), *Chitinophagales* (0.013% vs. 0.001%), and *Micrococcales* (0.012% vs. 0.007%) was higher under high forest management than under coppice (Figure 5a). In contrast, the relative abundance of the fungal order *Russulales* was higher under coppice management (0.10% vs. 0.06%) (Figure 5b).

**FIGURE 6:**
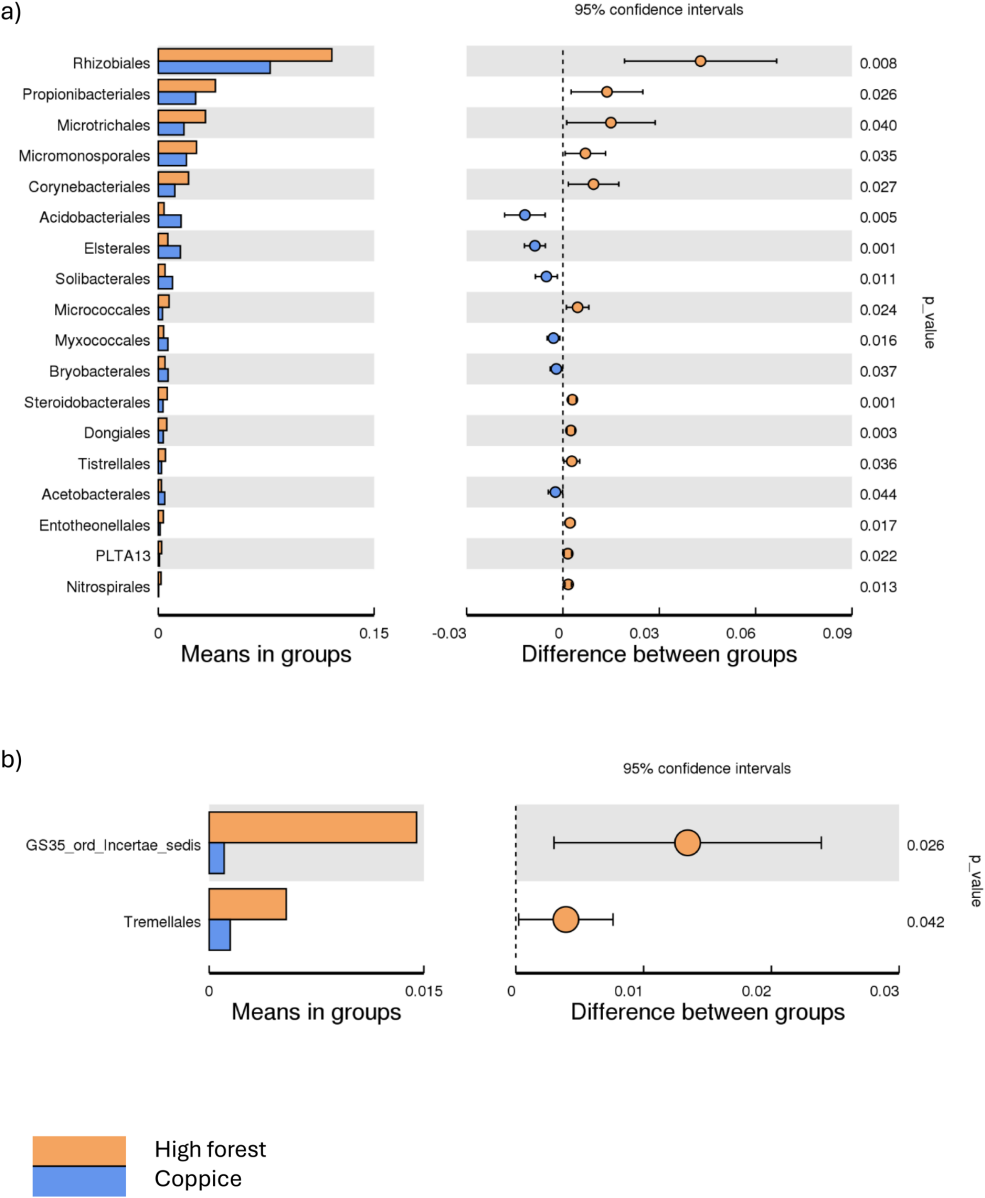
Barplots (left panel) showing the relative abundances (%) of soil (a) bacterial and (b) fungal orders in turkey oak forests managed by coppice and high forest. The right panel displays the difference in relative abundances of each order between the two management types (± lower and upper limits of 95% confidence interval).

In turkey oak forests, both bacterial and fungal communities showed higher relative abundances under high forest compared to coppice management. Bacterial taxa enriched under high forest included *Rhizobiales* (0.12% vs. 0.01%), *Propionibacteriales* (0.04% vs. 0.003%), *Microtrichales* (0.03 vs. 0.008%), *Micromonosporales* (0.03% vs. 0.003%), *Corynebacteriales* (0.007% vs. 0.005%) and *Micrococcales* (0.01% vs. 0.002%) (Figure 6a). Among fungi, the relative abundance of the orders *GS35_ord_Incertae_sedis* (0.015 vs. 0.001%) and *Tremellales* (0.005% vs. 0.001%) was higher under high forest than coppice (Figure 6b).

Here, we present the results for microbial community structure at the order taxonomic level for each forest system. Notably, the same trend in relative abundances was observed at both the class and family levels.

## 4 Discussion

Our results indicate that forest cover type and seasonality were the main factors shaping the soil microbial community functions. It is well established that season and plant species influence soil physico‐chemical properties and enzymatic activities (Eslaminejad et al. 2020; Luo et al. 2020). The separation between the two forests was attributable to the different forest cover types, which, through their input to the soil, differently affect the soil chemical–physical properties (i.e., organic matter and labile organic carbon) that lead, in turn, to different microbial communities (Picariello et al. 2021).

Seasonal effects on microbial metabolism were probably linked to changes in root exudates and leaf litter, driven by the emergence of new herbaceous and shrubby vegetation that colonised the plots during autumn and spring (based on observational data). In fact, the undergrowth was more abundant in these seasons compared to summer, supporting microbial metabolism. Total organic carbon also changed with the season, with higher average values in spring and summer, which may promote microbial metabolism. In contrast, dry summer conditions (average temperature 27°C, rainfall 12 mm, (Campania Region 2022)) resulted in lower enzymatic activities compared to other seasons.

Importantly, coppice management in either forest systems did not adversely affect the soil organic matter pool or microbial community activity, and consequently, the associated ecological functions. In fact, the careful forest management applied, with a 15‐year cutting cycle, supports the return of organic matter to the soil, facilitating the recovery of the soil microbial community (Picariello and De Nicola 2024).

The majority of changes in nutrient cycling occur in the initial years after cutting, as increased litter, plant residues, and root exudates contribute to organic matter accumulation and modify decomposition processes over the medium to long term (Pignataro et al. 2012). The recovery of the soil microbial community after coppicing is a dynamic process influenced by various climatic and management factors, that can extend well beyond the timeframe of our study (Ananbeh et al. 2019; Camponi et al. 2022).

In our study, the structure of the soil microbial communities, unlike their enzyme activities, was not influenced by the season in these Mediterranean ecosystems. However, other studies in areas under Mediterranean climate have reported seasonal changes in microbial composition: the soil fungal community in a reforested *Pinus pinaster* (Aiton) forest changed between the cold and moist months and the warm and dry months (Castaño et al. 2018); similarly, the microbial community structure, analyzed through phospholipid fatty acids, showed significant seasonal effects in three forests dominated by different pine species (Gazol et al. 2024).

Forest management did not affect soil microbial community function, but it affected the relative abundance of bacterial and fungal orders. This suggests that different microbial taxa may respond divergently to disturbance while maintaining similar ecological functions, highlighting the importance of functional redundancy in sustaining soil ecosystem services. Such resilience mechanisms have been reported in other forest ecosystems (Deng et al. 2016; Osburn et al. 2019; Jiang et al. 2024).

To our knowledge, there are few studies that distinguish the specific response of bacteria versus fungi to forest management practices (Panico et al. 2025), and many works have assumed that a change in the microbial community automatically entailed a functional change (Deng et al. 2016; Osburn et al. 2019; Kučera et al. 2021; Jiang et al. 2024). Our study highlights opposite responses of fungi and bacteria to management (coppice vs. high forest), contributing to the understanding of the dynamics between different microbial domains and demonstrating that the composition can vary without altering the enzymatic functions, suggesting functional resilience or ecological redundancy.

In beech forests, both soil fungi and bacteria showed a different response under different management practices. Factors such as understory vegetation, root depth and root exudation may have contributed to shaping soil fungal communities in coppice‐managed beech forest. Furthermore, the regular wood removal alters dead wood quality compared to unmanaged forests, influencing fungal communities. Specifically, *Russuales*, the most abundant order in the soil of coppice‐managed beech forests, belongs to the Basidiomycota kingdom. The higher soil labile carbon content in coppice‐managed beech with respect to high forest supports Basidiomycota, which produce enzymes such as alpha‐galactosidase, beta‐mannosidase, and xylosidase, crucial in the degradation of less recalcitrant organic matter, including plant polysaccharides (Manici et al. 2024).

Conversely, in turkey oak forests, the relative abundances of both soil bacterial and fungal taxonomic groups (at the order level) were higher in high forest management with respect to coppice. The most prevalent bacterial orders in high forest were *Rhizobiales*, involved in nitrogen fixation (Jones 2015), *Propionibacteriales*, key in fermentations (Johnson and Cummins 1972), and *Microtrichales*, which support plant growth and nutrient cycling (Lei et al. 2023). The dominant fungal order was *Tremellales*, of the *Tremellomycetes* class, known for decomposing resistant organic matter, including phenolic compounds (Freedman et al. 2015), although in turkey oak forests, we did not find significant differences in soil recalcitrant carbon between managements. In unmanaged areas where a high input of dead wood reaches the forest floor, we could suppose a higher soil recalcitrant carbon content, but likely the coppice system recovered after 15 years from disturbance, reaching and sometimes exceeding some of the chemical properties values measured under high forest.

Overall, our results suggest that the influence of forest management and seasonality is highly context‐dependent, being strongly modulated by forest type, meaning that the same management can lead to contrasting microbial responses in beech versus turkey oak forests. This context dependency highlights the importance of considering forest type as a key variable in sustainable forest planning, given that forest management has traditionally been evaluated in terms of timber productivity, rather than the broader ecosystem services supported by soil microbial communities (Ameray et al. 2021).

The parallel analysis of bacterial and fungal communities through high‐throughput sequencing, combined with enzyme activities and carbon utilisation patterns, provided a comprehensive view of the soil system and allowed us to highlight the contrasting responses of different microbial taxa under different forest systems. This integrative framework provides novel evidence for functional resilience and redundancy in forest soils and contributes to filling knowledge gaps on how microbial communities support ecosystem services under different management regimes in Mediterranean ecosystems.

## 5 Conclusions

The dominant tree species was the main factor shaping soil microbial communities in the studied forests. Forest management changed the relative abundances of microbial communities, but it did not remarkably affect microbial community functions and, thus, the associated ecosystem services.

Since the soil microbial community responded differently under the two management practices, particularly under beech compared to turkey oak, the type of forest should be taken into account when evaluating forest management. Future ecological studies focused on other forest types subjected to wood provision need to better understand these dynamics.

Although extrapolation to other forest ecosystems should be made with caution, our findings suggest that the mechanisms identified, particularly the stronger influence of vegetation cover relative to management, are likely to be relevant beyond Mediterranean systems, even if modulated by local soil and climatic conditions. From a management perspective, our results indicate that forest planning should explicitly account for dominant tree species, as they exert the primary control on soil microbial communities. Moreover, moderate coppicing cycles appear compatible with the maintenance of microbial functions, supporting the resilience of ecosystems and provision of their services. Specifically, we recommend that forest managers: (i) Consider dominant tree species when planning interventions, as vegetation cover exerts the strongest influence on microbial communities; (ii) adopt moderate coppicing cycles, as microbial functions show resilience after 15 years, suggesting that ecosystem services can be maintained; (iii) integrate microbial indicators into forest monitoring, since microbial structure may change even when functions remain stable, providing early‐warning signals of ecological shifts.

This study offers new insights into the key factors shaping soil microbial communities through the complex interactions between plant cover types, seasonality, and management practices. These findings are valuable for promoting sustainable forest management to support climate adaptation and enhance forest resilience.

## Author Contributions

**Enrica Picariello:** investigation, writing – original draft, writing – review and editing, software, formal analysis, data curation. **Flavia De Nicola:** funding acquisition, supervision, writing – review and editing, conceptualization.

## Funding

This work was supported by Fondo di Ricerca di Ateneo FRA 2023.

## Conflicts of Interest

The authors declare no conflicts of interest.

## Supporting information


**Figure S1:** Variance explained by (a) Dim1 and (b) Dim2 of the soil physico‐chemical variables and the relative abundance at order level processed by principal component analyses (PCA).

**Table S1:** Sum of Square, F model, R2 and P adjusted values of Pairwise tests calculated on all the measured physicochemical parameters and enzymatic activities. Asterisks indicate significant differences between management, season and forest system (*** *p* < 0.001, ** *p* < 0.01, * *p* < 0.05).

**Table S2:** (a) Water content (WC), pH and soil organic matter (SOM) (mean values ± s.e.) of beech and turkey oak soils. (b) Total organic carbon (total C), labile organic carbon (Cl) and recalcitrant organic carbon (Cr) (mean values ± s.e.) of beech and turkey oak soils.

**Table S3:** (a) F model, p adjusted values and significance of Three Way Repeated Measures ANOVA performed on water content (WC), soil organic matter (SOM) and pH, considering forest systems, forest management and season fixed factor. (b) F model, p and p adjusted values of Three Way Repeated Measures ANOVA performed on total organic C (Ctot), labile C and recalcitrant C, considering forest systems, forest management and season fixed factor.

## Data Availability

All data will be available on request. All raw data sets are publicly available in NCBI Sequence Read Archive (SRA) under project accession no PRJNA1354468—https://www.ncbi.nlm.nih.gov/sra/PRJNA1354468.
