# Supplementary material for: Unveiling the Hidden Drivers: How Vegetation Cover, Season and Forest Management Shape the Soil Microbial Community in Two Mediterranean Forest Ecosystems
Source: Environ Microbiol Rep. 2026 Mar 19;18(2):e70255. doi: 10.1111/1758-2229.70255 (PMC13053141; doi:10.1111/1758-2229.70255)

Supplementary figures

Fig S1. Variance explained by a) Dim1 and b) Dim2 of the soil physico-chemical variables and the relative abundance at order level processed by principal component analyses (PCA).

a)


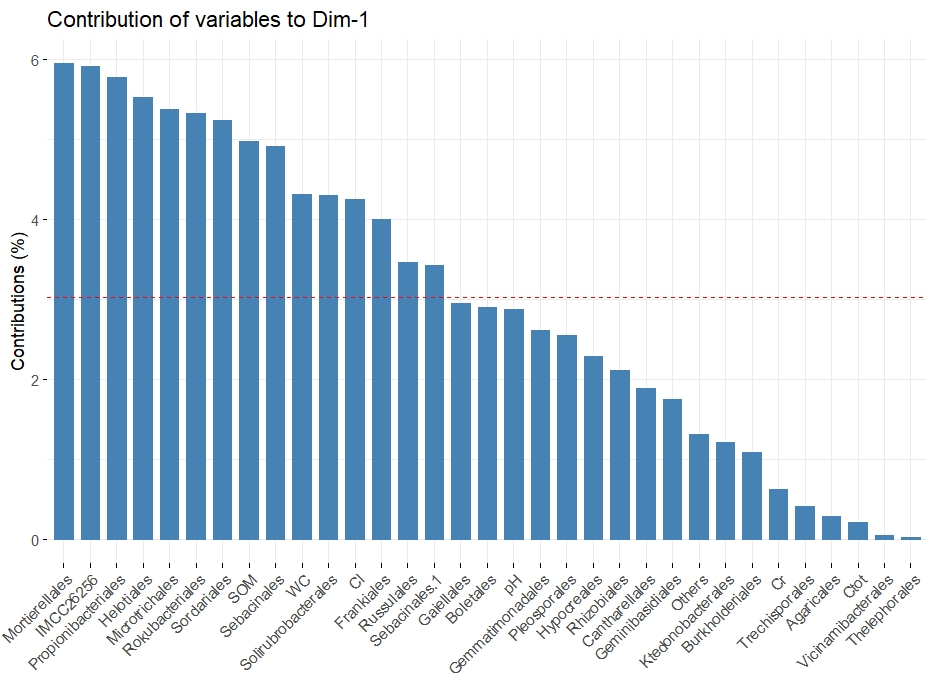


b)


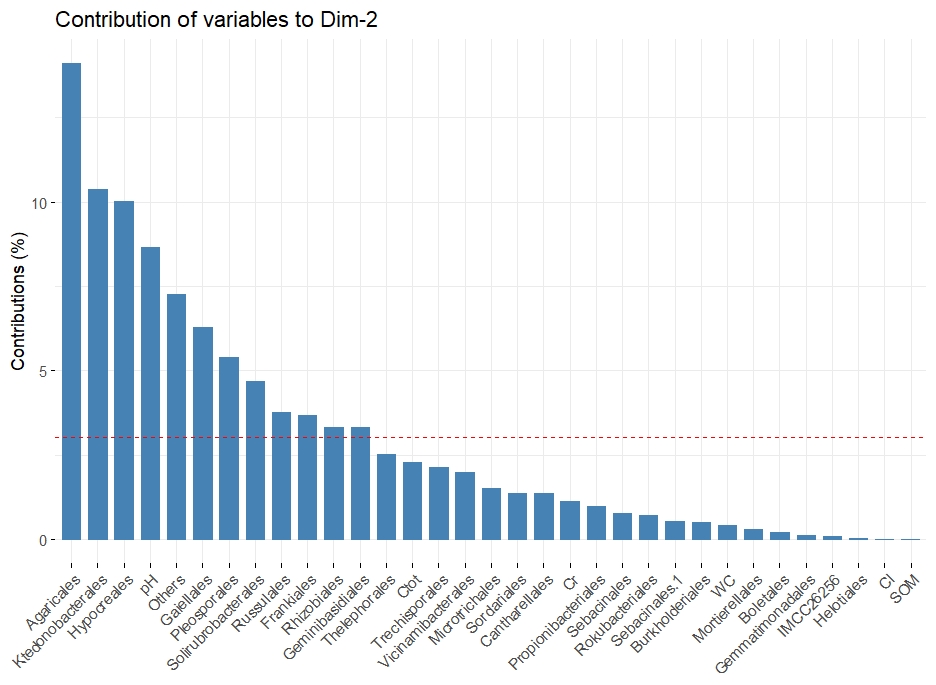

Supplement: Supplementary file 1 — Figure S1: Variance explained by (a) Dim1 and (b) Dim2 of the soil physico‐chemical variables and the relative abundance at order level processed by principal component analyses (PCA). [file EMI4-18-e70255-s002.docx]
