# Supplementary material for: Unveiling the Hidden Drivers: How Vegetation Cover, Season and Forest Management Shape the Soil Microbial Community in Two Mediterranean Forest Ecosystems
Source: Environ Microbiol Rep. 2026 Mar 19;18(2):e70255. doi: 10.1111/1758-2229.70255 (PMC13053141; doi:10.1111/1758-2229.70255)
Supplement: Supplementary file 2 — Table S1: Sum of Square, F model, R2 and P adjusted values of Pairwise tests calculated on all the measured physicochemical parameters and enzymatic activities. Asterisks indicate significant differences between management, season and forest system (*** p < 0.001, ** p < 0.01, * p < 0.05). [file EMI4-18-e70255-s003.docx]

**Table S1**. Sum of Square, F model, R2 and P adjusted values of Pairwise tests calculated on all the measured physicochemical parameters and enzymatic activities. Asterisks indicate significant differences between management, season and forest system (*** P<0.001, ** P<0.01, * P<0.05).

|  |  |  |  |  |  |
| --- | --- | --- | --- | --- | --- |
| **pairs** | **Sums Of Sqs** | **F Model** | **R2** | **P adjusted** | **sig** |
| **Forest system** |  |  |  |  |  |
| Beech *vs* Turkey oak | 0.434 | 17.562 | 0.276 | 0.001 | ** |
| **Season** |  |  |  |  |  |
| summer *vs* autumn | 0.506 | 38.634 | 0.637 | 0.006 | * |
| summer *vs*winter | 0.618 | 38.656 | 0.637 | 0.006 | * |
| summer *vs* spring | 0.090 | 7.858 | 0.263 | 0.006 | * |
| autumn *vs* winter | 0.044 | 2.070 | 0.086 | 0.708 |  |
| autumn *vs*spring | 0.199 | 11.897 | 0.351 | 0.006 | * |
| winter *vs* spring | 0.245 | 12.507 | 0.362 | 0.006 | * |
