# Supplementary material for: Unveiling the Hidden Drivers: How Vegetation Cover, Season and Forest Management Shape the Soil Microbial Community in Two Mediterranean Forest Ecosystems
Source: Environ Microbiol Rep. 2026 Mar 19;18(2):e70255. doi: 10.1111/1758-2229.70255 (PMC13053141; doi:10.1111/1758-2229.70255)
Supplement: Supplementary file 3 — Table S2: (a) Water content (WC), pH and soil organic matter (SOM) (mean values ± s.e.) of beech and turkey oak soils. (b) Total organic carbon (total C), labile organic carbon (Cl) and recalcitrant organic carbon (Cr) (mean values ± s.e.) of beech and turkey oak soils. [file EMI4-18-e70255-s001.docx]

Table S1a. Water content (WC), pH and soil organic matter (SOM) (mean values ± s.e.) of beech and turkey oak soils.

| **BEECH** |  |  |  |  |  |  |  |
| --- | --- | --- | --- | --- | --- | --- | --- |
| **Season** | **Management** | **WC (%)** | **se** | **pH** | **se** | **SOM (%)** | **se** |
| **autumn** | **coppice** | 44.71 | 1.50 | 5.61 | 0.13 | 27.65 | 1.37 |
| **autumn** | **high forest** | 45.00 | 0.91 | 6.56 | 0.18 | 25.91 | 1.11 |
| **spring** | **coppice** | 47.80 | 0.41 | 5.96 | 0.07 | 28.52 | 1.52 |
| **spring** | **high forest** | 44.72 | 0.85 | 6.27 | 0.16 | 25.59 | 0.37 |
| **summer** | **coppice** | 43.81 | 0.24 | 5.80 | 0.01 | 27.84 | 1.00 |
| **summer** | **high forest** | 39.00 | 0.60 | 6.23 | 0.05 | 22.50 | 0.62 |
| **winter** | **coppice** | 49.23 | 1.66 | 6.14 | 0.00 | 28.80 | 1.29 |
| **winter** | **high forest** | 46.59 | 0.51 | 6.34 | 0.14 | 25.01 | 0.81 |
|  |  |  |  |  |  |  |  |
| **TURKEY OAK** |  |  |  |  |  |  |  |
| **Season** | **Management** | **WC (%)** | **se** | **pH** | **se** | **SOM (%)** | **se** |
| **autumn** | **coppice** | 23.07 | 0.26 | 6.17 | 0.32 | 9.02 | 0.29 |
| **autumn** | **high forest** | 26.21 | 0.95 | 7.21 | 0.26 | 11.44 | 0.21 |
| **spring** | **coppice** | 29.29 | 1.73 | 6.37 | 0.26 | 10.32 | 1.42 |
| **spring** | **high forest** | 30.78 | 1.29 | 6.85 | 0.17 | 15.02 | 2.35 |
| **summer** | **coppice** | 12.76 | 0.23 | 6.64 | 0.05 | 15.63 | 0.22 |
| **summer** | **high forest** | 16.04 | 0.28 | 6.93 | 0.04 | 17.09 | 2.26 |
| **winter** | **coppice** | 28.36 | 1.29 | 6.52 | 0.27 | 9.76 | 1.41 |
| **winter** | **high forest** | 35.49 | 2.57 | 7.60 | 0.14 | 14.25 | 0.69 |

Table S1b. Total organic carbon (total C), labile organic carbon (Cl) and recalcitrant organic carbon (Cr) (mean values ± s.e.) of beech and turkey oak soils.

| **BEECH** |  |  |  |  |  |  |  |  |
| --- | --- | --- | --- | --- | --- | --- | --- | --- |
| **Season** | **Management** | **total C (mg C g^-1^ d.w.)** | **se** | **Cl (mg C g^-1^ d.w.)** | **se** | **Cr (mg C g^-1^ d.w.)** | **se** |  |
| **autumn** | **coppice** | 140.07 | 4.32 | 27.89 | 0.84 | 6.92 | 0.18 |  |
| **autumn** | **high forest** | 132.76 | 2.16 | 26.04 | 2.62 | 6.12 | 0.09 |  |
| **spring** | **coppice** | 247.37 | 7.41 | 44.88 | 8.41 | 10.67 | 0.84 |  |
| **spring** | **high forest** | 244.89 | 1.26 | 22.81 | 0.89 | 4.40 | 0.35 |  |
| **summer** | **coppice** | 265.35 | 6.20 | 23.03 | 0.40 | 5.35 | 0.53 |  |
| **summer** | **high forest** | 249.49 | 8.84 | 27.02 | 0.44 | 4.30 | 0.26 |  |
| **winter** | **coppice** | 126.65 | 2.00 | 38.95 | 2.67 | 5.69 | 0.38 |  |
| **winter** | **high forest** | 120.83 | 2.53 | 30.85 | 4.76 | 6.53 | 1.13 |  |
|  |  |  |  |  |  |  |  |  |
| **TURKEY OAK** | |  |  |  |  |  |  |  |
| **Season** | | **Management** | **total C (mg C g^-1^ d.w.)** | **se** | **Cl (mg C g^-1^ d.w.)** | **se** | **Cr (mg C g^-1^ d.w.)** | **se** |
| **autumn** | **coppice** | 107.49 | 31.99 | 21.61 | 0.94 | 3.93 | 0.14 |  |
| **autumn** | **high forest** | 89.96 | 1.96 | 20.71 | 0.58 | 4.16 | 0.26 |  |
| **spring** | **coppice** | 149.87 | 14.60 | 13.33 | 0.92 | 7.68 | 0.37 |  |
| **spring** | **high forest** | 195.51 | 23.98 | 14.95 | 0.26 | 7.13 | 0.51 |  |
| **summer** | **coppice** | 284.31 | 6.95 | 16.96 | 0.56 | 2.94 | 0.03 |  |
| **summer** | **high forest** | 288.30 | 26.83 | 15.47 | 0.97 | 3.35 | 0.31 |  |
| **winter** | **coppice** | 76.01 | 8.84 | 14.21 | 0.69 | 7.44 | 0.48 |  |
| **winter** | **high forest** | 95.86 | 5.74 | 14.56 | 1.50 | 5.63 | 0.93 |  |
