# Supplementary material for: Unveiling the Hidden Drivers: How Vegetation Cover, Season and Forest Management Shape the Soil Microbial Community in Two Mediterranean Forest Ecosystems
Source: Environ Microbiol Rep. 2026 Mar 19;18(2):e70255. doi: 10.1111/1758-2229.70255 (PMC13053141; doi:10.1111/1758-2229.70255)
Supplement: Supplementary file 4 — Table S3: (a) F model, p adjusted values and significance of Three Way Repeated Measures ANOVA performed on water content (WC), soil organic matter (SOM) and pH, considering forest systems, forest management and season fixed factor. (b) F model, p and p adjusted values of Three Way Repeated Measures ANOVA performed on total organic C (Ctot), labile C and recalcitrant C, considering forest systems, forest management and season fixed factor. [file EMI4-18-e70255-s004.docx]

Table S2a. F model, p adjusted values and significance of Three Way Repeated Measures ANOVA performed on water content (WC), soil organic matter (SOM) and pH, considering forest systems, forest management and season fixed factor.

|  | **Effect** | **F** | **p** | **p<.05** |
| --- | --- | --- | --- | --- |
| **WC** |  |  |  |  |
|  | management | 0.526 | 5.44E-01 |  |
|  | season | 98.458 | 1.71E-05 | * |
|  | system | 1849.167 | 5.40E-04 | * |
|  | management:season | 5.151 | 4.30E-02 | * |
|  | management:system | 44.637 | 2.20E-02 | * |
|  | season:system | 44.574 | 1.70E-04 | * |
|  | management:season:system | 0.784 | 5.45E-01 |  |
| **SOM** |  |  |  |  |
|  | management | 0.008 | 0.939 |  |
|  | season | 4.72 | 0.051 |  |
|  | system | 250.161 | 0.004 | * |
|  | management:season | 1.71 | 0.264 |  |
|  | management:system | 15.689 | 0.058 |  |
|  | season:system | 7.173 | 0.021 | * |
|  | management:season:system | 0.546 | 0.669 |  |
| **pH** |  |  |  |  |
|  |  |  |  |  |
|  | management | 161.002 | 0.006 | * |
|  | season | 2.704 | 0.139 |  |
|  | system | 55.747 | 0.017 | * |
|  | management:season | 27.541 | 0.000661 | * |
|  | management:system | 1.287 | 0.374 |  |
|  | season:system | 2.262 | 0.182 |  |
|  | management:season:system | 2.51 | 0.156 |  |

Statistic, p, p adjusted values and significance level of Pairwise tests calculated on water content (WC), soil organic matter (SOM) and pH.

|  |  |  | **statistic** | **p** | **p.adj** | **p.adj.signif** |
| --- | --- | --- | --- | --- | --- | --- |
| **WC** |  |  |  |  |  |  |
|  | autumn | spring | -3.561267 | 4.00E-03 | 0.027 | * |
|  | autumn | summer | 5.563864 | 1.69E-04 | 0.001 | ** |
|  | autumn | winter | -3.996301 | 2.00E-03 | 0.013 | * |
|  | spring | summer | 5.90041 | 1.03E-04 | 0.000618 | *** |
|  | spring | winter | -1.820712 | 9.60E-02 | 0.575 | ns |
|  | summer | winter | -6.401615 | 5.07E-05 | 0.000304 | *** |
|  | beech | turkey oak | 15.39223 | 1.33E-13 | 1.33E-13 | **** |
|  | coppice | high forest | -0.6244744 | 0.538 | 0.538 | ns |
| **SOM** |  |  |  |  |  |  |
|  | beech | turkey oak | 12.40367 | 1.14E-11 | 1.14E-11 | **** |
|  | coppice | high forest | 0.09620105 | 0.924 | 0.924 | ns |
| **pH** |  |  |  |  |  |  |
|  | beech | turkey oak | -8.21852 | 2.69E-08 | 2.69E-08 | **** |
|  | coppice | high forest | -6.794745 | 6.26E-07 | 6.26E-07 | **** |

Note: Asterisks indicate significant differences in each Pairwise test (****p < .0001***p < .001, **p < .01, *p < .05).

Table S2b. F model, p and p adjusted values of Three Way Repeated Measures ANOVA performed on total organic C (Ctot), labile C and recalcitrant C, considering forest systems, forest management and season fixed factor.

|  | **Effect** | **F** | **p** | **p<.05** |
| --- | --- | --- | --- | --- |
| **Ctot** |  |  |  |  |
|  | management | 0.049 | 8.46E-01 |  |
|  | season | 110.425 | 1.22E-05 | * |
|  | system | 29.5 | 3.20E-02 | * |
|  | management:season | 2.087 | 2.03E-01 |  |
|  | management:system | 2.174 | 2.78E-01 |  |
|  | season:system | 8.727 | 1.30E-02 | * |
|  | management:season:system | 1.266 | 3.67E-01 |  |
| **labile C** |  |  |  |  |
|  | management | 14.756 | 0.062 |  |
|  | season | 1.574 | 0.291 |  |
|  | system | 212.952 | 0.005 | * |
|  | management:season | 2.666 | 0.142 |  |
|  | management:system | 7.475 | 0.112 |  |
|  | season:system | 7.906 | 0.017 | * |
|  | management:season:system | 5.756 | 0.034 | * |
| **recalcitrant C** |  |  |  |  |
|  |  |  |  |  |
|  | management | 7.993 | 0.106 |  |
|  | season | 23.35 | 0.001 | * |
|  | system | 4.955 | 0.156 |  |
|  | management:season | 21.93 | 0.001 | * |
|  | management:system | 8.925 | 0.096 |  |
|  | season:system | 14.935 | 0.003 | * |
|  | management:season:system | 28.25 | 0.000616 | * |

Statistic, p, p adjusted values and significance level of Pairwise tests calculated on total organic C (Ctot), labile C and recalcitrant C.

|  |  |  | **statistic** | **p** | **p.adj** | **p.adj.signif** |
| --- | --- | --- | --- | --- | --- | --- |
| **total organic C** |  |  |  |  |  |  |
|  | autumn | spring | -6.698018 | 3.39E-05 | 2.03E-04 | *** |
|  | autumn | summer | -11.519241 | 1.77E-07 | 1.06E-06 | **** |
|  | autumn | winter | 1.481782 | 1.66E-01 | 9.96E-01 | ns |
|  | spring | summer | -3.639175 | 4.00E-03 | 2.30E-02 | * |
|  | spring | winter | 12.732491 | 6.31E-08 | 3.79E-07 | **** |
|  | summer | winter | 13.431493 | 3.62E-08 | 2.17E-07 | **** |
|  | beech | turkey oak | 3.046919 | 0.006 | 0.006 | ** |
|  | coppice | high forest | -0.3720661 | 0.713 | 0.713 | ns |
| **labile C** |  |  |  |  |  |  |
|  | autumn | spring | 0.01985942 | 0.985 | 1 | ns |
|  | autumn | summer | 3.13394851 | 0.01 | 0.057 | ns |
|  | autumn | winter | -0.21465809 | 0.834 | 1 | ns |
|  | spring | summer | 0.89625177 | 0.389 | 1 | ns |
|  | spring | winter | -0.22441836 | 0.827 | 1 | ns |
|  | summer | winter | -1.61052124 | 0.136 | 0.816 | ns |
|  | beech | turkey oak | 6.280466 | 2.08E-06 | 2.08E-06 | **** |
|  | coppice | high forest | 1.813866 | 0.083 | 0.083 | ns |
| **recalcitrant C** |  |  |  |  |  |  |
|  | autumn | spring | -3.073118 | 1.10E-02 | 0.064 | ns |
|  | autumn | summer | 7.18726 | 1.78E-05 | 0.000107 | *** |
|  | autumn | winter | -1.718043 | 1.14E-01 | 0.684 | ns |
|  | spring | summer | 5.416177 | 2.11E-04 | 0.001 | ** |
|  | spring | winter | 1.389795 | 1.92E-01 | 1 | ns |
|  | summer | winter | -4.205956 | 1.00E-03 | 0.009 | ** |
|  | beech | turkey oak | 2.099922 | 0.047 | 0.047 | * |
|  | coppice | high forest | 2.377661 | 0.026 | 0.026 | * |

Note: Asterisks indicate significant differences in each Pairwise test (****p < .0001***p < .001, **p < .01, *p < .05).
